# Specific PCR primer designed from genome data for rapid detection of *Fusarium oxysporum* f. sp. *cubense* tropical race 4 in the Cavendish banana

**DOI:** 10.1371/journal.pone.0313358

**Published:** 2024-12-02

**Authors:** Shunsuke Nozawa, Dan Charlie Joy Pangilinan, G. Alvindia Dionisio, Kyoko Watanabe

**Affiliations:** 1 School of Agriculture, Tamagawa University, Machida, Tokyo, Japan; 2 Philippine Center for Postharvest Development and Mechanization, Nueva Ecija, Philippines; ICAR - Indian Agricultural Research Institute, INDIA

## Abstract

Fusarium wilt caused by *Fusarium oxysporum* f. sp. *cubense* (Foc) tropical race 4 (TR4) severely affects banana production worldwide. Thus, specific PCR primers have been developed to rapidly diagnose and monitor Foc TR4-related fusarium wilt outbreaks in bananas. However, evaluation of these primers revealed room for improvement in the accuracy. This study aimed to design highly specific PCR primers based on genome data for Foc TR4 downloaded from the National Center for Biotechnology Information database. The specificity of the primers was assessed using Foc TR4, Foc races 1 and 2, and 15 other formae speciales strains. The utility of the primers was verified by correctly detecting Foc TR4 in 7 out of 86 isolates of *Fusarium* spp. obtained from banana farms in the Philippines. The primers allowed for rapid detection in experimentally diseased tissues. We concluded that this novel primer set enables the simplified diagnosis of fusarium wilt caused by Foc TR4 in bananas.

## Introduction

Bananas are an important cash crop in Southeast Asia, Africa, and Latin America. According to FAOSTAT, banana production reached 1.25 million tons in 2021. However, banana production has been threatened by banana wilt disease caused by *Fusarium oxysporum* f. sp. *cubense* (Foc), a soil-borne pathogen. In the 20th century, the disease caused by Foc race 1 eradicated thousands of hectares of the Gros Michel banana in Central America. In response to this, the resistant variety, Cavendish banana was discovered and this variety became the dominant commercial banana worldwide. However, since its 1989 outbreak in a Cavendish plantation in Taiwan [1, 2], fusarium wilt caused by Foc tropical race 4 TR4 (syn. *Fusarium odoratissimum*) has severely affected banana production worldwide. Since Foc TR4 produces durable chlamydospores that can survive in the soil for more than a decade without a host, during which susceptible varieties cannot be successfully replanted [3, 4]. Given their importance in food production, worldwide monitoring of disease outbreaks has been implemented.

Foc TR4 has been identified through pathogenicity testing of susceptible varieties and determining vegetative compatibility groups (VCGs) using nitrate non-utilizing (nit) mutants [5]. However, pathogenicity and VCG testing may take 1–2 months, and VCGs cannot be identified without reference strains. A recent study showed that although it is difficult to find differences in morphology from other races, Foc TR4 is independent of other lineages including Foc race 1 and Foc subtropical race 4 (STR4), based on phylogenetic analysis using four loci: calmodulin (*cmdA*), RNA polymerase second largest subunit (*rpb2*), translation elongation factor 1-alpha (*tef1*), and β-tubulin 2 (*tub2*) regions [6]. Therefore, Foc TR4 can be distinguished from other races by phylogenetic analysis. However, this method also requires >1 month for isolation, purification, DNA extraction, polymerase chain reaction (PCR), sequencing, and phylogenetic analysis. Thus, conventional detection methods are unsuitable for the rapid diagnosis or investigation of disease outbreaks.

To address the current gap in the field, Foc TR4-specific PCR primers have been designed. Dita et al. [7] and Li et al. [8] accomplished this using the intergenic spacer region of the rDNA cluster (IGS), while Li et al. [9] and Carvalhais et al. [10] designed primers based on pathogen-related genes encoded by xylem 1 and W2987, respectively. However, because conserved regions, such as the IGS, cannot accurately distinguish differences [11], false-positive rates are high [12]. Using the primers designed for pathogen-related genes by Li et al. [9] and Carvalhais et al. [10], Yang et al. [12] revealed that some Foc TR4 strains were not detected and disease incidence may be underestimated. In this study, we aimed to develop a specific primer set based on the genome data for the Foc TR4-specific site. This primer set represents a promising epidemiological tool for the rapid diagnosis of fusarium wilt.

## Materials and methods

### Phylogenetic analysis using genome data

We performed a genome-scale phylogenetic analysis based on a concatenation approach to confirm that the Foc TR4 strains were monophyletic and determine the phylogenetic relationships between Foc TR4 and other races and formae speciales. The genome data of 32 formae speciales (93 strains, including Foc TR4) were retrieved from the National Center for Biotechnology Information (NCBI) database (S1 Table) and used as an ingroup. *F*. *fujikuroi* (IMI58289) was retrieved from the NCBI database and used as an outgroup. Gene predictions were performed with Augustus v.3.3.3 [13] with the following parameter: “—species = fusarium <genomic data>”. Orthology inference was performed by reciprocal BLAST analysis (BLASTP) with BLAST 2.9.0+ software (*e* ≤ 1e-05) [14, 15]. Multiple alignments for each orthologous gene were conducted using Clustal Omega v.1.2.2 [16] with the default settings at the translated amino acid sequence level. To select genes comprising >1,000 bp after alignment, we trimmed gap-including sites with trimAl v.1.2 [17] and counted the remaining sites using PhyKIT v.1.2.0 (https://jlsteenwyk.com/PhyKIT/ [18]). Phylogenetic trees were constructed using a concatenation approach. The concatenated alignment of DNA data from all gene sets (4,595 genes) was analyzed using RAxML v.7.0.4 [19] under the GTRGAMMA model with 100 bootstrap replicates. A phylogenetic tree was drawn using iTOL v.6 (https://itol.embl.de/).

### Primer design

We used the genome data for 21 strains of Foc TR4 and 93 strains of 32 different formae speciales (the same dataset used to construct the genome-scale phylogenetic trees) to explore Foc TR4-specific genes. Gene prediction and orthology inferences were performed as described in **Phylogenetic analysis using genome data**. Genes found only in Foc TR4, excluding BC2-4, were selected as candidates. BLAST analyses were conducted using the DNA sequence data of candidate genes against the genome data to confirm their detection in genome regions where they were not previously predicted. Primers were designed using Primer3Plus (https://www.primer3plus.com/) with the default parameters for melting temperature, GC content, and primer length. The presence of the sequence was verified through a BLAST search on the NCBI website (https://blast.ncbi.nlm.nih.gov/Blast.cgi?PROGRAM=blastn&PAGE_TYPE=BlastSearch&LINK_LOC=blasthome).

### Assessment of primer set specificity

To compare the accuracy between our primer set and those designed by Carvalhais et al. [10], Dita et al. [7], Li et al. [9], and Li et al. [8], the primers were also tested under reported PCR conditions (S2 Table). Foc TR4 isolates (2718M; S1 Fig) that confirmed its pathogenicity against the Cavendish variety and (S2 Fig), Foc race1, Foc race2, and 15 other formae speciales (25 isolates, Table 1) deposited at the National Agricultural and Food Research Organization (NARO) Genetic Resources Research Centre (Kumamoto, Japan) were used for PCR. Template DNA for PCR was obtained from the mycelia of each isolate grown on potato dextrose agar for 7–10 d using a modified CTAB method [20], as described by Nozawa et al. [21]. PCR was performed using a 10 μL PCR mixture containing 7 μL distilled water, 1 μL 10× Ex Taq buffer with MgCl_2_, 0.8 μL dNTP (10 mM each), 0.1 μL of each primer (50 μM), 0.05 μL Ex Taq DNA polymerase (5 U μL^−1^, Takara, Tokyo, Japan), and 1.0 μL of DNA template. Amplification of the partial 18S rRNA gene, ITS1, 5.8S rRNA gene, ITS2, and partial 28S rRNA gene [internal transcribed spacer (ITS) region] was performed to confirm successful DNA extraction. The PCR conditions described by White et al. [22] were used to amplify the ITS region. The thermal cycling program for primers 13721F and 13712R consisted of an initial denaturing step of 5 min at 95 °C, followed by 35 cycles of 10 s at 95 °C, 5 s at 56 °C, and 10 s at 72 °C, with a final extension step of 3 min at 72 °C.

**Table 1:** Strains of pathogenic *F*. *oxysporum* and results of PCR using primers for specific detection of FocTR4.

Strain	f. sp. (race)	Host	Primer sets				
			13712F 13712R (this study)	FocTR4F FocTR4R (Dita et al. 2010)	W2987F W2987R (Li et al., 2013b)	SIX1a-266-F SIX1a-266-R (Carvalhais et al., 2019)	VCG01213 16F1 VCG01213 16R2 (Li et al., 2013a)
2718M	cubense (TR4)	banana	+	+	-	+	+
race1	cubence (race 1)	banana	-	-	-	-	+
race2	cubence (race 2)	banana	-	+	-	-	+
MAFF 103008	lagenariae	bottle gourd	-	+	-	-	-
MAFF 103036	lycopersici	tomato	-	-	-	-	+
MAFF 103051	melogenae	eggplant	-	+	-	-	+
MAFF 103054	cucumerinam	cucumber	-	+	-	-	+
MAFF 103059	spinaciae	spinach	-	-	-	-	+
MAFF 235105	tulipae	tulip	-	-	-	-	-
MAFF 235154	not determine	rice	-	+	-	-	+
MAFF 237022	not determine	taro	-	+	-	-	-
MAFF 240102	tanaceti	feverfew	-	+	-	-	+
MAFF 240327	rapae	tatsoi	-	-	-	-	-
MAFF 240804	not determine	bitter gourd	-	+	-	-	+
MAFF 240805	not determine	bitter gourd	-	+	-	-	+
MAFF 241054	adzukicala	azuki bean	-	+	-	-	+
MAFF 243255	lactucae	lettuce	-	+	-	-	+
MAFF 243476	not determine	delphinium	-	+	-	-	+
MAFF 305543	niveum	watermelon	-	-	-	-	+
MAFF 305544	melonis	melon	-	+	-	-	+
MAFF 305606	batatas	sweet potato	-	+	+	+	-
MAFF 305608	niveum	watermelon	-	-	-	-	+
MAFF 305937	radicis-lycopersici	tomato	-	+	-	-	+
MAFF 306313	not determine	taro	-	+	-	-	+
MAFF 727508	cacumerinum	cucumber	-	+	-	-	+
MAFF 744004	cacumerinum	cucumber	-	+	-	-	+
MAFF 744088	lactucae	lettuce	-	-	-	-	+
MAFF 306716	cubence	banana	-	+	-	-	+

‘+’ indicates that the relevant strain was detected with the primers.

‘-’ indicated that the relevant strain was not detected with the primers.

### Investigating Foc TR4 in isolates from the Philippines

Symptomatic plants were collected from banana farms in Mindanao in 2022 to obtain banana isolates. Discolored vascular tissues of the pseudostem were cut into 5 × 5 mm pieces that were then sterilized with 0.6% (v/v) sodium hypochlorite for 1 min, washed with sterilized water, dried with sterilized paper, and placed on a water–agar (WA) plate. Hyphae that emerged on the WA were transferred onto potato dextrose agar plates to produce conidia for monoculture. The obtained 86 isolates of *Fusarium* spp. were used for subsequent experiments.

PCR amplification of the 86 isolates was conducted using primer sets 13712F and 13712R. A 200-bp amplicon was confirmed by gel electrophoresis. We confirmed that the detected isolates were clustered with Foc TR4. First, to select isolates belonging to the *F*. *oxysporum* species complex Foc TR4, molecular phylogenetic analysis was performed on the 86 isolates and ex-type strains of *Fusarium* species based on the ITS region (S1 Dataset).

We performed a molecular phylogenetic analysis of the 21 isolates clustered with the *F*. *oxysporum* species complex based on the concatenated data for *cmdA*, *rpb2*, *tef1*, and *tub2* regions as previously described [6] to reveal the phylogenetic positions of the isolates (S2 Dataset). We selected 20 species, consisting of 54 strains in the *F*. *oxysporum* species complex, reported by Lombard et al. [6]; *F*. *foetens* (CBS120665) and *F*. *udum* (CBS130302) were used as outgroups (Table 2). Alignment of each sequence dataset was performed using ClustalW in BioEdit v.7.2 [23]. The aligned datasets were combined for phylogenetic analyses using neighbor-joining (NJ), maximum-likelihood (ML), and maximum-parsimony (MP) algorithms in MEGA10 [24]. Gap-including sites were treated as missing data. The reliability of the internal branches in the trees was evaluated through a 1,000 replicate bootstrap analysis [25].

**Table 2:** Strains and Genebank accession number using phylogenetic analysis.

Species	Strain no.	Accession no.			
		*cmdA*	*rpb2*	*tef1*	*tub2*
*F*. *callistephi*	CBS187.53	MH484693	MH484875	MH484966	MH485057
	CBS115423	MH484723	MH484905	MH484996	MH485087
*F*. *carminascens*	CBS144739	MH484752	MH484934	MH485025	MH485116
	CBS144740	MH484753	MH484935	MH485026	MH485117
	CBS144741	MH484754	MH484936	MH485027	MH485118
*F*. *contaminatum*	CBS111552	MH484718	MH484900	MH484991	MH485082
	CBS114899	MH484719	MH484901	MH484992	MH485083
	CBS117461	MH484729	MH484911	MH485002	MH485093
*F*. *cugenangense*	CBS620.72	MH484697	MH484879	MH484970	MH485061
	CBS130304	MH484739	MH484921	MH485012	MH485103
	CBS130308	MH484738	MH484920	MH485011	MH485102
*F*. *curvatum*	CBS247.61	MH484694	MH484876	MH484967	MH485058
	CBS238.94	MH484711	MH484893	MH484984	MH485075
	CBS141.95	MH484712	MH484894	MH484985	MH485076
*F*. *duoseptatum*	CBS102026	MH484714	MH484896	MH484987	MH485078
*F*. *elaeidis*	CBS217.49	MH484688	MH484870	MH484961	MH485052
	CBS218.49	MH484689	MH484871	MH484962	MH485053
	CBS255.52	MH484692	MH484874	MH484965	MH485056
*F*. *fabacearum*	CBS144742	MH484756	MH484938	MH485029	MH485120
	CBS144743	MH484757	MH484939	MH485030	MH485121
	CBS144744	MH484758	MH484940	MH485031	MH485122
*F*. *foetens*	CBS120665	MH484736	MH484918	MH485009	MH485100
*F*. *glycines*	CBS176.33	MH484686	MH484868	MH484959	MH485050
	CBS214.49	MH484687	MH484869	MH484960	MH485051
	CBS200.89	MH484706	MH484888	MH484979	MH485070
*F*. *gossypinum*	CBS116611	MH484725	MH484907	MH484998	MH485089
	CBS116612	MH484726	MH484908	MH484999	MH485090
	CBS116613	MH484727	MH484909	MH485000	MH485091
*F*. *hoodiae*	CBS132474	MH484747	MH484929	MH485020	MH485111
	CBS132476	MH484748	MH484930	MH485021	MH485112
	CBS132477	MH484749	MH484931	MH485022	MH485113
*F*. *languescens*	CBS645.78	MH484698	MH484880	MH484971	MH485062
	CBS646.78	MH484699	MH484881	MH484972	MH485063
	CBS413.90	MH484708	MH484890	MH484981	MH485072
*F*. *libertatis*	CBS144748	MH484750	MH484932	MH485023	MH485114
	CBS144747	MH484751	MH484933	MH485024	MH485115
	CBS144749	MH484762	MH484944	MH485035	MH485126
*F*. *nirenbergiae*	CBS129.24	MH484682	MH484864	MH484955	MH485046
	CBS149.25	MH484683	MH484865	MH484956	MH485047
	CBS840.88	MH484705	MH484887	MH484978	MH485069
*F*. *odoratissimum*	CBS794.70	MH484696	MH484878	MH484969	MH485060
	CBS102030	MH484716	MH484898	MH484989	MH485080
	CBS102030	MH484740	MH484922	MH485013	MH485104
*F*. *oxysporum*	CBS130310	MH484690	MH484872	MH484963	MH485054
	CBS221.49	MH484771	MH484953	MH485044	MH485135
	CBS144134	MH484772	MH484954	MH485045	MH485136
*F*. *pharetrum*	CPC25822	MH484769	MH484951	MH485042	MH485133
	CBS144750	MH484770	MH484952	MH485043	MH485134
*F*. *trachichlamydosporum*	CBS144751	MH484715	MH484897	MH484988	MH485079
*F*. *triseptatum*	CBS102028	MH484691	MH484873	MH484964	MH485055
	CBS258.50	MH484728	MH484910	MH485001	MH485092
	CBS116619	MH484734	MH484916	MH485007	MH485098
*F*. *udum*	CBS130302	MH484684	MH484866	MH484957	MH485048
*F*. *veterinarium*	CBS177.31	MH484717	MH484899	MH484990	MH485081
	CBS109898	MH484730	MH484912	MH485003	MH485094
	CBS117787	MH484731	MH484913	MH485004	MH485095

### Detection in plant tissues

PCR amplification was conducted using DNA extracted from banana tissues to demonstrate the diagnosis of diseased tissues. A pathogenicity test was conducted to obtain diseased banana tissues as previously described [21]. Tissues (0.2 g) from corms with dark brown to black lesions and healthy tissues (without inoculation) were used for DNA extraction using the CTAB method. PCR amplification was conducted three times under the conditions described in **Assessment of primer set specificity**. DNA templates extracted from healthy bananas and sterile water were used as negative controls, and mycelia of Foc TR4 were used as positive controls. DNA extraction was confirmed by gel electrophoresis.

## Results

### Phylogenetic relationship between Foc TR4 and other races as well as formae speciales

**Fig 1:**
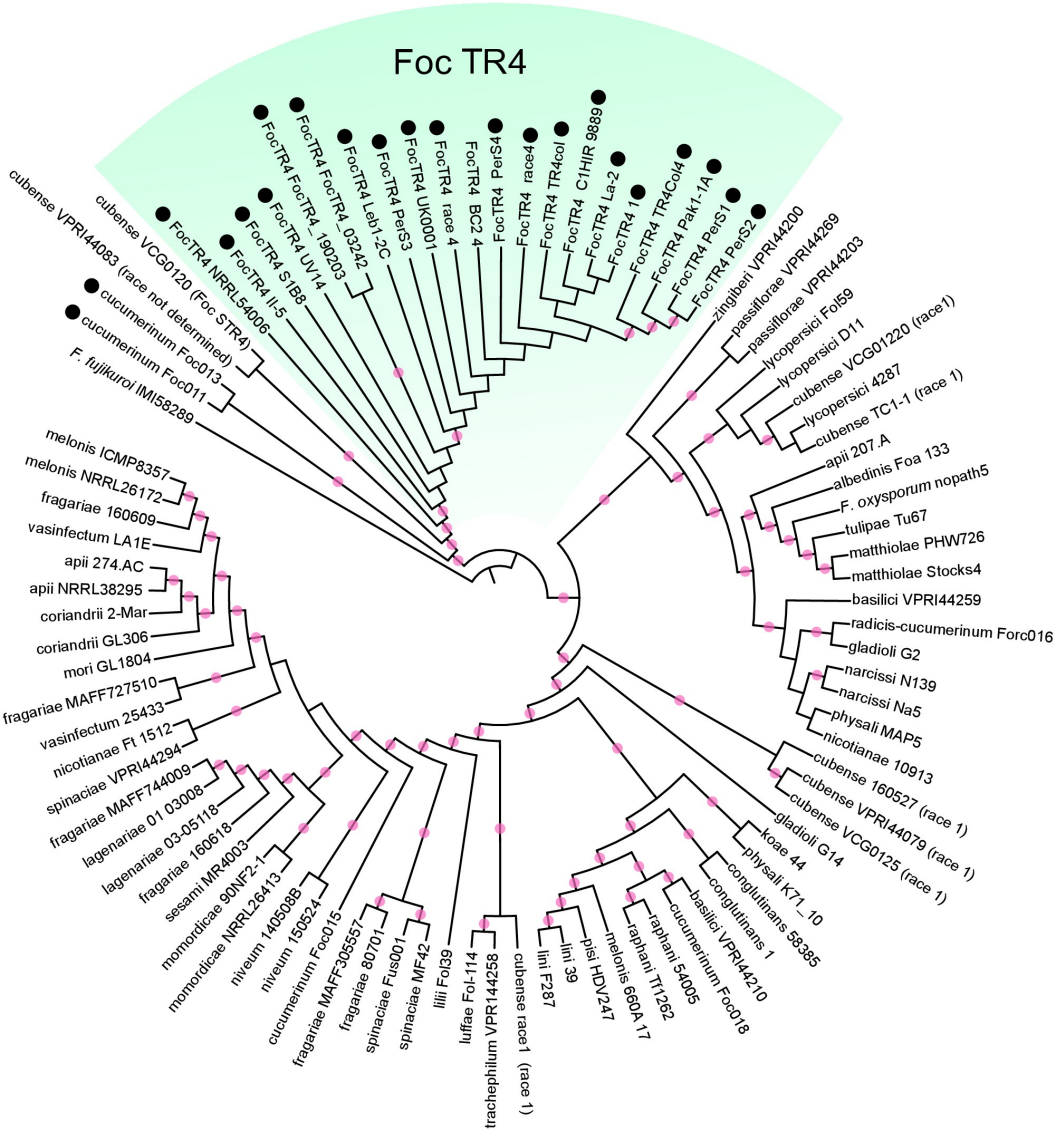
Phylogenetic trees based on the concatenated DNA sequence data for 4,595 orthologous genes of the protein-coding region. Red circles on internal branches indicate 90% bootstrap (BS) values. Isolates with a black circle carry gene 13712, from which primers were designed. The green-highlighted clade is a Foc TR4 clade.

To confirm that Foc TR4 is monophyletic, we constructed a molecular phylogenetic tree based on the genome data deposited on NCBI. We identified 4,595 orthologous genes through reciprocal BLAST searches. The alignment length, number of variable sites, and number of parsimony-informative sites in the DNA data were 8,356,161 bp, 923,458, and 345,950, respectively. The ML tree showed that the 21 Foc TR4 strains were monophyletic, supported with a 100% bootstrap value (Fig 1).

### Primer design

Among the Foc TR4-specific genes from the genome data, gene 13712 was found to be best for primer design, though it was absent in strain BC2-4 (Foc TR4) and present in strains Foc011 and Foc013 (f. sp. *cucumerinum*, Fig 1). Strains Foc011 and Foc013 with gene 13712 were monophyletic (100% bootstrap value), and polyphyletic with the Foc TR4 clade. These two strains separated into a lineage consisting of f. sp. *cubense* subtropical race 4 (STR4, VCG0120) and f. sp. *cubense* (VPRI44083, race not determined).

**Fig 2:**
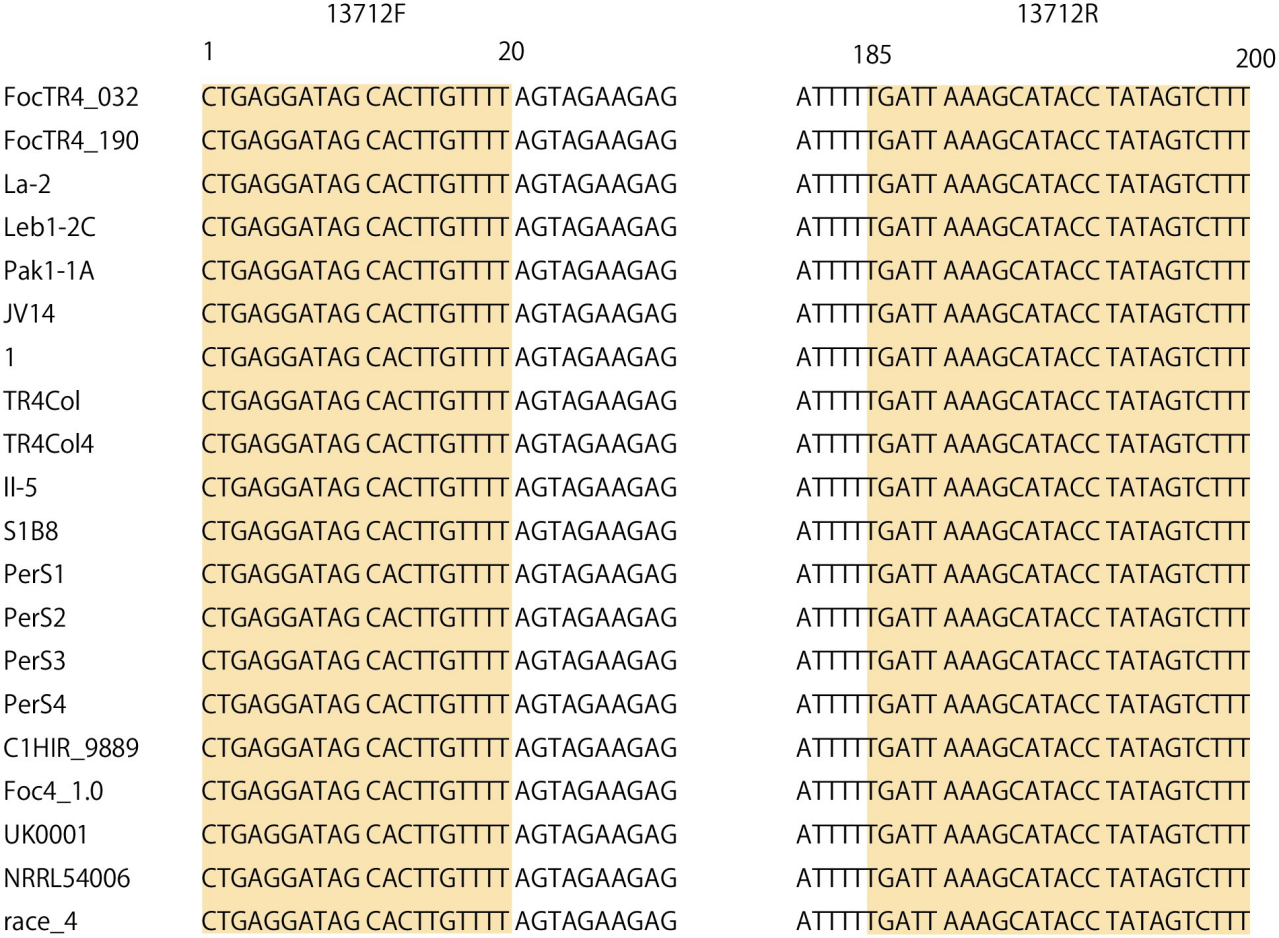
Alignment of primer sitting on gene 13712 of Foc TR4 strains. Highlighted sites are primer sites.

The primer set 13712F (5′-CTG AGGA TAG CAC TTG TTT T-3′) and 13712R (5′-AAA GAC TAT AGG TAT GCT TTA ATC A-3′), which produces a 201-bp PCR amplicon (S1 Data), was designed based on the DNA sequence of gene 13712. For a forward primer 13712F, GC content is 40%, and the annealing temperature is 53.2°C. For a reverse primer13712R, GC content is 26.9%, and the annealing temperature is 54.6°C. The primer sites were completely conserved among the 20 Foc TR4 strains, except for the BC2-4 strain (Fig 2).

### Assessment of primers 13712F and 13712R

We performed PCR amplification of a 200-bp DNA fragment from the Foc TR4 strain using primers 13712F and 13712R (Table 1, S3 Fig). This is consistent with the expected length from the genome data. To confirm the identity of the amplified fragment, the sequence was compared to the target region obtained from the genome data. The resulting fragment sequence was 100% identical to the corresponding sequence in the genome data. No PCR DNA amplicons were amplified from other *F*. *oxysporum* formae speciales, including *adzukicala*, *batatas*, *cubense*, *cucumerinam*, *lactucae*, *lagenariae*, *lycopersici*, *melogenae*, *melonis*, *niveum*, *radicis-lycopersici*, *rapae*, *spinaciae*, *tanaceti*, and *tulipae*, or strains with no formae speciales assignment but showing pathogenicity against bitter gourd, delphinium, rice, and taro. In contrast, the primers designed previously for the detection of Foc TR4 by Dita et al. [7], Carvalhais et al. [10], and Li et al. [9] amplified 18, 1, and 21 strains other than Foc TR4. The primers designed by Li et al. [8] did not amplify the 452-bp target region of Foc TR4. Therefore, the primer set designed in this study showed more specificity for Foc TR4 than previously reported primers.

### Detection of Foc TR4 from banana farms

**Fig 3:**
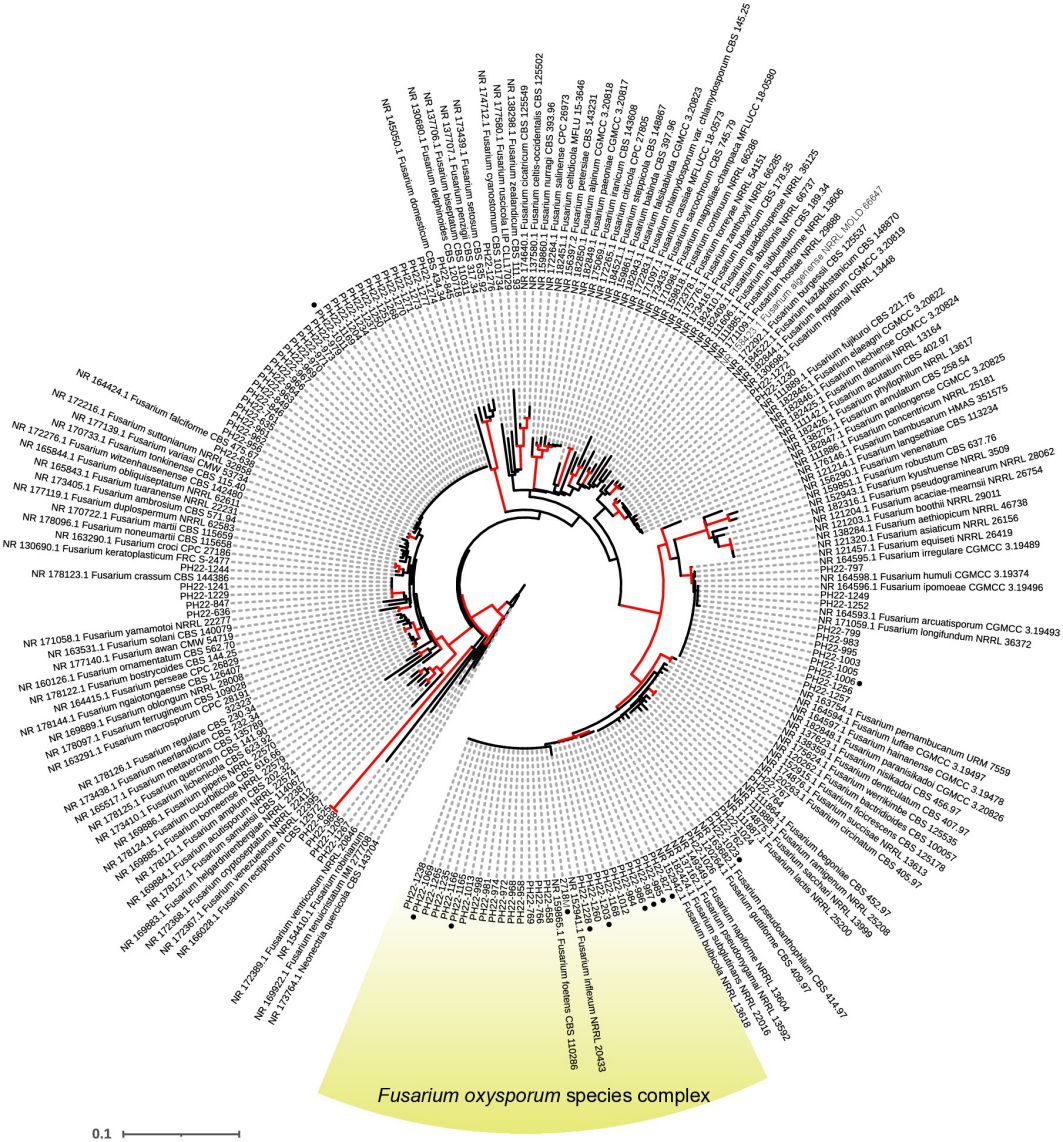
Phylogenetic relationships of isolates in this study and known species inferred using the sequence for the ITS region. Evolutionary history was inferred using the NJ method. The tree is drawn to scale, with branch lengths in the same units as those of the evolutionary distances used to infer the phylogenetic tree. The evolutionary distances were computed using the p-distance method and are expressed as the number of base differences per site. There were a total of 472 positions in the final dataset. Evolutionary analyses were conducted in MEGA10. Isolates with black dots were detected using the primer set designed in this study. Red branches have >70% bootstrap values.

**Fig 4:**
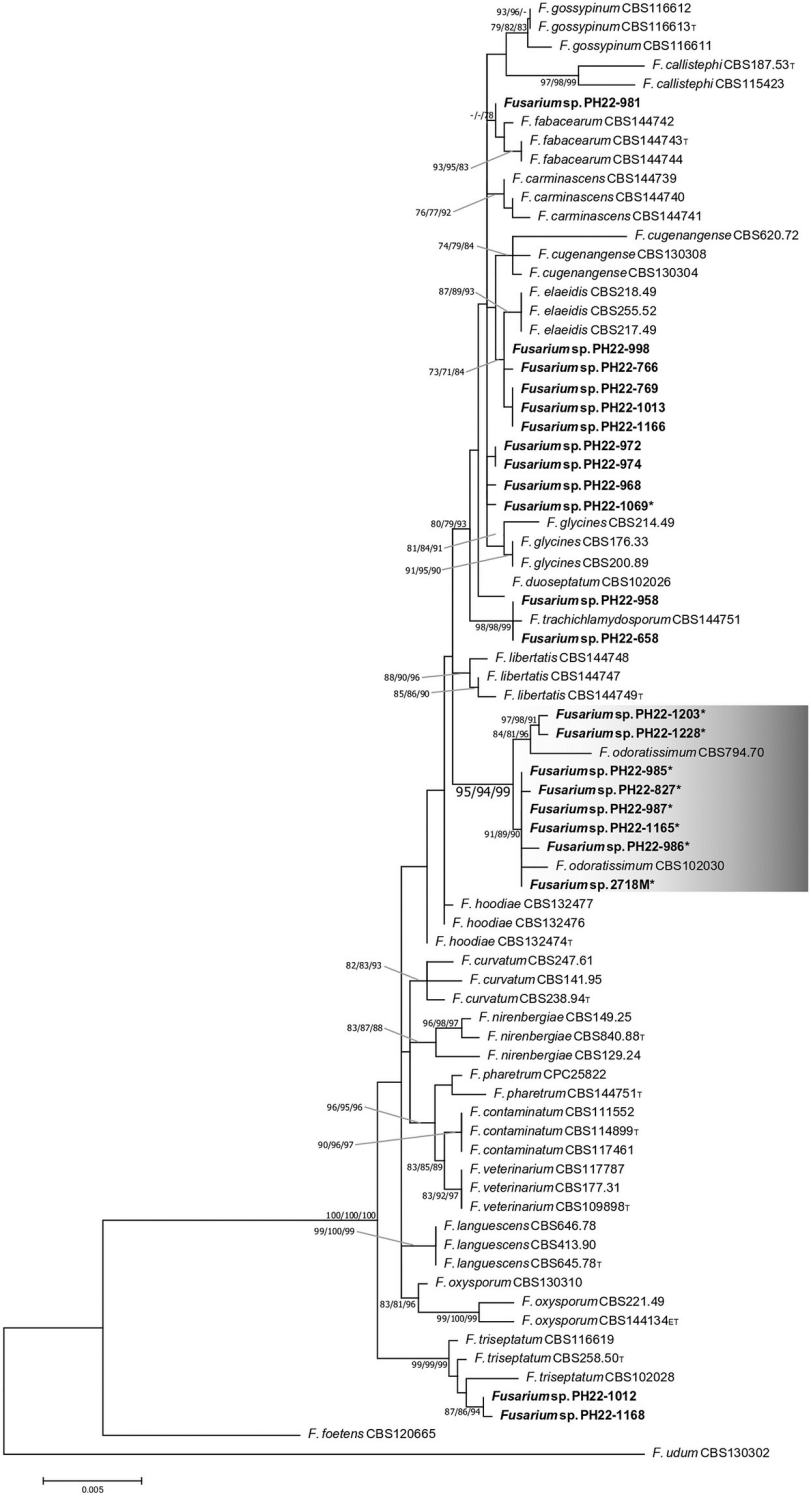
Phylogenetic relationships of isolates in this study and known species inferred using the concatenated data for the four loci (2,378 bp), *cmdA* (423 bp), *rpb2* (877 bp), *tef1* (620 bp), and *tub2* (458 bp). Evolutionary history was inferred using the ML method. The values accompanying internal branches are bootstrap values (ML/NJ/MP, ≥70%). -, absence of node; T, ex-type strain; ET, ex-epitype strain. Isolates with black dots were detected using the primer set designed in this study.

Based on the ITS region, 21 of the 86 isolates collected from banana farms in the Philippines were clustered with the *F*. *oxysporum* species complex (92% bootstrap value) in the phylogenetic tree (Fig 3). Molecular phylogenetic analysis of the concatenated data for the four loci (2,378 bp), *cmdA* (423 bp), *rpb2* (877 bp), *tef1* (620 bp), and *tub2* (458 bp) identified seven isolates forming a monophyletic clade with *F*. *odoratissimum* strains, which are considered to be Foc TR4 (ML/NJ/MP = 94%/97%/99% bootstrap value; Fig 4). These isolates were effectively detected through PCR using the primer set 13712F and 13712R (Table 3). Other isolates were clustered with *F*. *elaeidis* (5 isolates; ML/NJ/MP = 73%/71%/84%), *F*. *fabacearum* (one isolate; ML/NJ/MP = -/-/78%), *F*. *trachichlamydosporum* (two isolates; ML/NJ/MP = 98%/98%/99%), and *F*. *triseptatum* (two isolates; ML/NJ/MP = 99%/99%/99%). Four isolates are independent of known species and closely related to *F*. *glycines*. One isolate, PH22-1069, of those, and three isolates, PH22-1006, PH22-1011, and PH22-1023, from other species complexes (not determined; Fig 3) were detected using this primer set were not Foc TR4 (Figs 3 and 4, S4 Fig).

**Table 3:** Results of PCR detection using primer set 13712F/13712R from isolates in the Philippines, their phylogenetic positions and location.

Isolates	Detection	Phylogenetic position	Location
PH22-0625	-	*Fusarium* sp. (not *F*. *oxysporum* species complex)	Balila, Lantapan, Bukidnon
PH22-0635	-	*Fusarium* sp. (not *F*. *oxysporum* species complex)	Midland, Valencia City, Bukidnon
PH22-0636	-	*Fusarium* sp. (not *F*. *oxysporum* species complex)	Midland, Valencia City, Bukidnon
PH22-0638	-	*Fusarium* sp. (not *F*. *oxysporum* species complex)	Midland, Valencia City, Bukidnon
PH22-0658	-	cf. *F*. *trachichlamydosporum*	Valencia City, Bukidnon
PH22-0761	-	*Fusarium* sp. (not *F*. *oxysporum* species complex)	Tibal-og (Poblacion), Sto. Tomas, Davao del Norte
PH22-0762	-	*Fusarium* sp. (not *F*. *oxysporum* species complex)	Tibal-og (Poblacion), Sto. Tomas, Davao del Norte
PH22-0764	-	*Fusarium* sp. (not *F*. *oxysporum* species complex)	Tibal-og (Poblacion), Sto. Tomas, Davao del Norte
PH22-0766	-	cf. *F*. *elaeidis*	Tibal-og (Poblacion), Sto. Tomas, Davao del Norte
PH22-0767	-	*Fusarium* sp. (not *F*. *oxysporum* species complex)	Tibal-og (Poblacion), Sto. Tomas, Davao del Norte
PH22-0769	-	cf. *F*. *elaeidis*	Tibal-og (Poblacion), Sto. Tomas, Davao del Norte
PH22-0797	-	*Fusarium* sp. (not *F*. *oxysporum* species complex)	Sto. Tomas, Davao del Norte
PH22-0799	-	*Fusarium* sp. (not *F*. *oxysporum* species complex)	Sto. Tomas, Davao del Norte
PH22-0827	+	Foc TR4 (cf. *F*. *odoratissimum*)	Davao Coastal Road, Mabini, Davao De Oro
PH22-0845	-	*Fusarium* sp. (not *F*. *oxysporum* species complex)	Davao Coastal Road, Mabini, Davao De Oro
PH22-0846	-	*Fusarium* sp. (not *F*. *oxysporum* species complex)	Davao Coastal Road, Mabini, Davao De Oro
PH22-0847	-	*Fusarium* sp. (not *F*. *oxysporum* species complex)	Davao Coastal Road, Mabini, Davao De Oro
PH22-0849	-	*Fusarium* sp. (not *F*. *oxysporum* species complex)	Davao Coastal Road, Mabini, Davao De Oro
PH22-0956	-	*Fusarium* sp. (not *F*. *oxysporum* species complex)	Finca Verde, Tugbok, Davao City, Davao del Sur
PH22-0958	-	cf. *F*. *trachichlamydosporum*	Finca Verde, Tugbok, Davao City, Davao del Sur
PH22-0961	-	*Fusarium* sp. (not *F*. *oxysporum* species complex)	Finca Verde, Tugbok, Davao City, Davao del Sur
PH22-0962	-	*Fusarium* sp. (not *F*. *oxysporum* species complex)	Finca Verde, Tugbok, Davao City, Davao del Sur
PH22-0963	-	*Fusarium* sp. (not *F*. *oxysporum* species complex)	Finca Verde, Tugbok, Davao City, Davao del Sur
PH22-0964	-	*Fusarium* sp. (not *F*. *oxysporum* species complex)	Finca Verde, Tugbok, Davao City, Davao del Sur
PH22-0966	-	*Fusarium* sp. (not *F*. *oxysporum* species complex)	Finca Verde, Tugbok, Davao City, Davao del Sur
PH22-0967	-	*Fusarium* sp. (not *F*. *oxysporum* species complex)	Finca Verde, Tugbok, Davao City, Davao del Sur
PH22-0968	-	*Fusarium* sp. (*F*. *oxysporum* species complex)	Finca Verde, Tugbok, Davao City, Davao del Sur
PH22-0969	-	*Fusarium* sp. (not *F*. *oxysporum* species complex)	Finca Verde, Tugbok, Davao City, Davao del Sur
PH22-0970	-	*Fusarium* sp. (not *F*. *oxysporum* species complex)	Finca Verde, Tugbok, Davao City, Davao del Sur
PH22-0971	-	*Fusarium* sp. (not *F*. *oxysporum* species complex)	Finca Verde, Tugbok, Davao City, Davao del Sur
PH22-0972	-	*Fusarium* sp. (*F*. *oxysporum* species complex)	Finca Verde, Tugbok, Davao City, Davao del Sur
PH22-0973	-	*Fusarium* sp. (not *F*. *oxysporum* species complex)	Finca Verde, Tugbok, Davao City, Davao del Sur
PH22-0974	-	*Fusarium* sp. (*F*. *oxysporum* species complex)	Finca Verde, Tugbok, Davao City, Davao del Sur
PH22-0979	-	*Fusarium* sp. (not *F*. *oxysporum* species complex)	Finca Verde, Tugbok, Davao City, Davao del Sur
PH22-0981	-	aff. *F*. *fabacearum*	Finca Verde, Tugbok, Davao City, Davao del Sur
PH22-0983	-	*Fusarium* sp. (not *F*. *oxysporum* species complex)	Finca Verde, Tugbok, Davao City, Davao del Sur
PH22-0984	-	*Fusarium* sp. (not *F*. *oxysporum* species complex)	Finca Verde, Tugbok, Davao City, Davao del Sur
PH22-0985	+	Foc TR4 (cf. *F*. *odoratissimum*)	Finca Verde, Tugbok, Davao City, Davao del Sur
PH22-0986	+	Foc TR4 (cf. *F*. *odoratissimum*)	Finca Verde, Tugbok, Davao City, Davao del Sur
PH22-0987	+	Foc TR4 (cf. *F*. *odoratissimum*)	Finca Verde, Tugbok, Davao City, Davao del Sur
PH22-0987	-	*Fusarium* sp. (not *F*. *oxysporum* species complex)	Finca Verde, Tugbok, Davao City, Davao del Sur
PH22-0988	-	*Fusarium* sp. (not *F*. *oxysporum* species complex)	Finca Verde, Tugbok, Davao City, Davao del Sur
PH22-0995	-	*Fusarium* sp. (not *F*. *oxysporum* species complex)	Finca Verde, Tugbok, Davao City, Davao del Sur
PH22-0998	-	cf. *F*. *elaeidis*	Finca Verde, Tugbok, Davao City, Davao del Sur
PH22-1003	-	*Fusarium* sp. (not *F*. *oxysporum* species complex)	Finca Verde, Tugbok, Davao City, Davao del Sur
PH22-1005	-	*Fusarium* sp. (not *F*. *oxysporum* species complex)	Finca Verde, Tugbok, Davao City, Davao del Sur
PH22-1006	+	*Fusarium* sp. (not *F*. *oxysporum* species complex)	Finca Verde, Tugbok, Davao City, Davao del Sur
PH22-1011	+	*Fusarium* sp. (not *F*. *oxysporum* species complex)	Finca Verde, Tugbok, Davao City, Davao del Sur
PH22-1012	-	cf. *F*. *triseptatum*	Finca Verde, Tugbok, Davao City, Davao del Sur
PH22-1013	-	cf. *F*. *elaeidis*	Finca Verde, Tugbok, Davao City, Davao del Sur
PH22-1023	-	*Fusarium* sp. (not *F*. *oxysporum* species complex)	Finca Verde, Tugbok, Davao City, Davao del Sur
PH22-1024	-	*Fusarium* sp. (not *F*. *oxysporum* species complex)	Finca Verde, Tugbok, Davao City, Davao del Sur
PH22-1026	-	*Fusarium* sp. (not *F*. *oxysporum* species complex)	Finca Verde, Tugbok, Davao City, Davao del Sur
PH22-1069	+	*Fusarium* sp. (*F*. *oxysporum* species complex)	Tamayong, Davao City, Davao del Sur
PH22-1165	+	cf. *F*. *triseptatum*	Biao, Guianga, Tugbok, Davao City, Davao del Sur
PH22-1166	-	cf. *F*. *elaeidis*	Biao, Guianga, Tugbok, Davao City, Davao del Sur
PH22-1168	-	cf. *F*. *triseptatum*	Biao, Guianga, Tugbok, Davao City, Davao del Sur
PH22-1169	+	*Fusarium* sp. (not *F*. *oxysporum* species complex)	Biao, Guianga, Tugbok, Davao City, Davao del Sur
PH22-1203	+	Foc TR4 (cf. *F*. *odoratissimum*)	Biao, Guianga, Tugbok, Davao City, Davao del Sur
PH22-1204	-	*Fusarium* sp. (not *F*. *oxysporum* species complex)	Biao, Guianga, Tugbok, Davao City, Davao del Sur
PH22-1205	-	*Fusarium* sp. (not *F*. *oxysporum* species complex)	Biao, Guianga, Tugbok, Davao City, Davao del Sur
PH22-1228	+	Foc TR4 (cf. *F*. *odoratissimum*)	Biao, Guianga, Tugbok, Davao City, Davao del Sur
PH22-1229	-	*Fusarium* sp. (not *F*. *oxysporum* species complex)	Biao, Guianga, Tugbok, Davao City, Davao del Sur
PH22-1230	-	*Fusarium* sp. (not *F*. *oxysporum* species complex)	Biao, Guianga, Tugbok, Davao City, Davao del Sur
PH22-1234	-	*Fusarium* sp. (not *F*. *oxysporum* species complex)	Biao, Guianga, Tugbok, Davao City, Davao del Sur
PH22-1235	-	*Fusarium* sp. (not *F*. *oxysporum* species complex)	Biao, Guianga, Tugbok, Davao City, Davao del Sur
PH22-1237	-	*Fusarium* sp. (not *F*. *oxysporum* species complex)	Biao, Guianga, Tugbok, Davao City, Davao del Sur
PH22-1238	-	*Fusarium* sp. (not *F*. *oxysporum* species complex)	Biao, Guianga, Tugbok, Davao City, Davao del Sur
PH22-1241	-	*Fusarium* sp. (not *F*. *oxysporum* species complex)	Biao, Guianga, Tugbok, Davao City, Davao del Sur
PH22-1244	-	*Fusarium* sp. (not *F*. *oxysporum* species complex)	Biao, Guianga, Tugbok, Davao City, Davao del Sur
PH22-1249	-	*Fusarium* sp. (not *F*. *oxysporum* species complex)	Biao, Guianga, Tugbok, Davao City, Davao del Sur
PH22-1250	-	*Fusarium* sp. (not *F*. *oxysporum* species complex)	Biao, Guianga, Tugbok, Davao City, Davao del Sur
PH22-1251	-	*Fusarium* sp. (not *F*. *oxysporum* species complex)	Biao, Guianga, Tugbok, Davao City, Davao del Sur
PH22-1252	-	*Fusarium* sp. (not *F*. *oxysporum* species complex)	Biao, Guianga, Tugbok, Davao City, Davao del Sur
PH22-1256	-	*Fusarium* sp. (not *F*. *oxysporum* species complex)	Biao, Guianga, Tugbok, Davao City, Davao del Sur
PH22-1257	-	*Fusarium* sp. (not *F*. *oxysporum* species complex)	Biao, Guianga, Tugbok, Davao City, Davao del Sur
PH22-1260	-	*Fusarium* sp. (not *F*. *oxysporum* species complex)	Biao, Guianga, Tugbok, Davao City, Davao del Sur
PH22-1265	-	*Fusarium* sp. (not *F*. *oxysporum* species complex)	Biao, Guianga, Tugbok, Davao City, Davao del Sur
PH22-1267	-	*Fusarium* sp. (not *F*. *oxysporum* species complex)	Biao, Guianga, Tugbok, Davao City, Davao del Sur
PH22-1268	-	*Fusarium* sp. (not *F*. *oxysporum* species complex)	Biao, Guianga, Tugbok, Davao City, Davao del Sur
PH22-1270	-	*Fusarium* sp. (not *F*. *oxysporum* species complex)	Biao, Guianga, Tugbok, Davao City, Davao del Sur
PH22-1271	-	*Fusarium* sp. (not *F*. *oxysporum* species complex)	Biao, Guianga, Tugbok, Davao City, Davao del Sur
PH22-1272	-	*Fusarium* sp. (not *F*. *oxysporum* species complex)	Biao, Guianga, Tugbok, Davao City, Davao del Sur
PH22-1273	-	*Fusarium* sp. (not *F*. *oxysporum* species complex)	Biao, Guianga, Tugbok, Davao City, Davao del Sur
PH22-1274	-	*Fusarium* sp. (not *F*. *oxysporum* species complex)	Biao, Guianga, Tugbok, Davao City, Davao del Sur
PH22-1276	-	*Fusarium* sp. (not *F*. *oxysporum* species complex)	Biao, Guianga, Tugbok, Davao City, Davao del Sur
PH22-1277	-	*Fusarium* sp. (not *F*. *oxysporum* species complex)	Biao, Guianga, Tugbok, Davao City, Davao del Sur

‘+’ indicates that the strain was detected with the primers.

‘-’ indicated that the strain was not detected with the primers.

### Detection in tissues

**Fig 5:**
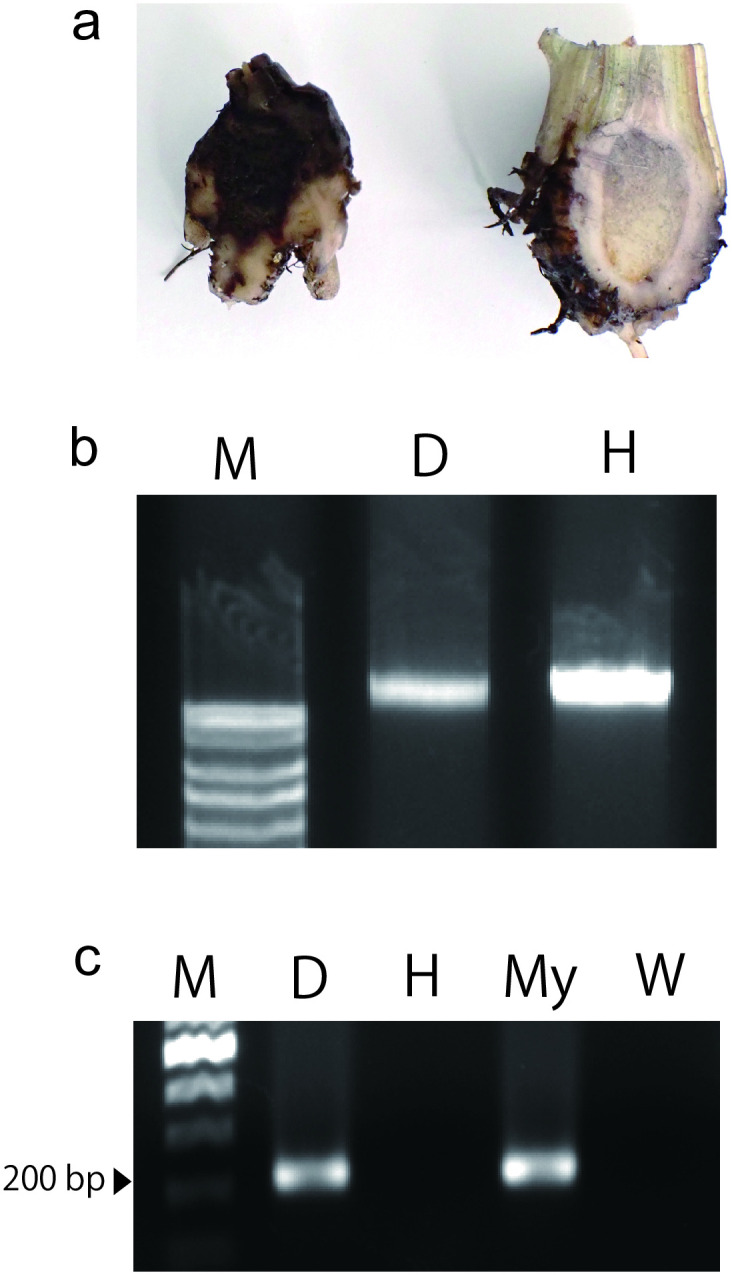
Detection in plant tissues by PCR amplification. **a** Diseased (left) and healthy (right) corms of dwarf cavendish banana with and without inoculation of Foc TR4 (2718M). **b** Extracted DNA from healthy and diseased tissues. Lane M: 1 kb DNA ladder; Lane D: genome DNA from diseased tissue; Lane H: genome DNA from healthy tissue. **c** Detection of PCR amplicons using DNA templates from healthy and diseased tissues and mycelia of Foc TR4 (2718M). Lane M: 100 bp DNA ladder. Lane D: PCR amplicon from diseased tissue. Lane H: PCR amplicon from healthy tissue. Lane My: PCR amplicon from mycelia. Lane W: Negative control with ddH_2_O.

The 200-bp PCR amplicons were only obtained from diseased tissues experimentally infected with Foc TR4 and Foc TR4 mycelia (Fig 5). No PCR amplicons were obtained from healthy tissues or water.

## Discussion

Fusarium wilt caused by Foc TR4 seriously threatens the productivity of the global banana industry. Rapid detection is important to prevent the spread of pathogens in the absence of resistant varieties. In this study, we designed more accurate and specific PCR primers for Foc TR4 than the previously developed primers. This study explored the genes specifically possessed by Foc TR4 from the genome data of 93 *F*. *oxysporum* strains and identified gene 13712 on which to base the primer set. Primers were validated by PCR using the Foc TR4 strain and other formae speciales (Table 1, S3 Fig). Compared to the primers developed by Dita et al. [7], Carvalhais et al. [10], Li et al. [8], and Li et al. [9], which did not detect Foc TR4 or detected other formae speciales, the primers designed in this study detected only Foc TR4 (Table 1, S3 Fig). These results indicate that the PCR primer set 13712F and 13712R is more accurate than previously reported primers.

We verified that the primers designed in this study effectively detected Foc TR4 among the 86 isolates of *Fusarium* spp. collected from banana fields in the Philippines based on the monophyletic classification of the race [6]. The PCR detection results identified seven strains that were monophyletic with Foc TR4, with no strains remaining undetected, at least as false negatives. In contrast, four of the 86 strains were detected as false positives. This may be because the target gene was horizontally transferred or this region, which was inherited from a common ancestor, remained intact in strains other than Foc TR4. However, based on our comparison of primer specificities by PCR trials including other formae speciales, the primer set 13712F and 13712R offered better detection (Table 1; S1 Fig), although not complete, because some isolates (4 out of 79 non-Foc TR4 strains) that are from fields and not Foc TR4 were detected. Furthermore, our primers successfully detected Foc TR4 in diseased plant tissue (Fig 5) and are expected to reduce the time and effort required for diagnosis.

As more regions and high-quality genome data, such as at the chromosomal level, become available for a wider range of lineages, so does the availability of appropriate sequences for designing Foc TR4-specific PCR primers. As with the genome data, further investigation into relevant strains is required to confirm the reliability of the designed primers. In this study, primer specificity was evaluated on 18 formae speciales, including Foc TR4 and other formae speciales strains. However, >100 *F*. *oxysporum* formae speciales have been reported up to August 2018 [26]. Since it is not realistic to evaluate primer specificity for all of them, further evaluation will be needed across various future studies on banana fusarium wilt.

Given the rapid evolution of plant pathogens, monitoring should be performed not only for Foc TR4 but also other potentially harmful pathogens. Therefore, pathogen detection using specific PCR primers should be performed periodically according to up-to-date classification systems and diagnosed based on Koch’s principles.

## Supporting information

S1 Fig Morophology of 2718M (*Fusarium oxysporum* f. sp. *cubense*). (PDF)

S2 Fig The result of pathogenicity test of *F*. *oxysporum* f. sp. *cubence* tropical race 4 (2718M) to bananas (cv. dwarf Cavendish). (PDF)

S3 Fig PCR detection of Foc TR4 using specific primers sets 13712F/13712R (this study), Foc TR4F/Foc TR4R [7], W2987F/W2987R [10], SIX1a-266-F/SIX1a-266-R [9], and VCG01213 16F/VCG01213 16R [8]. Lane M, marker; lane 1, Foc TR4; lane 2, Foc race 1; lane 3, Foc race 2; lane 4, MAFF103008; lane 5, MAFF235154; lane 6, MAFF240805; lane 7, MAFF237022; lane 8, MAFF243476; lane 9, MAFF727508; lane 10, MAFF306716; lane 11, MAFF744004; lane 12, MAFF305544; lane 13, MAFF240804; lane 14, MAFF243255; lane 15, MAAFF241054; lane 16, MAFF306313; lane 17, MAFF305937; lane 18, MAFF103036; lane 19, MAFF235105; lane 20, MAFF744088; lane 21, MAFF103054; lane 22, MAFF305606; lane 23, MAFF240327; lane 24, MAFF103059; lane 25, MAFF103051; lane 26, MAFF240102; lane 27, MAFF305543; lane 28, MAFF305608; lane C, ddH2O.
(PDF)

S4 Fig PCR analysis of *Fusarium* spp. using primer set 13712F/13712R. Isolate names are shown in each lane. The first lane of each panel (M) shows the marker sizes using the ExcelBand^™^ 100 bp DNA ladder (SMOBIO). The upper and lower panels show the amplification results for the ITS and 13712 regions, respectively. Isolates with red characters indicate amplification using primer set 13712F/13712R. Isolates with red dots indicate that isolates were identified as Foc TR4.
(PDF)

S1 Table GenBank accession numbers for genome data. (XLSX)

S2 Table List of FocTR4 specific primers used in this study. (XLSX)

S1 Data Sequence of an amplicon obtained from 2718M by PCR using primer set 13712F/13712R. (TXT)

S1 Dataset Sequence data sets for ITS. (TXT)

S2 Dataset Sequence data sets for *cmdA*, *rpb2*, *tef1*, and *tub2*. (ZIP)
