# Supplementary material for: Specific PCR primer designed from genome data for rapid detection of Fusarium oxysporum f. sp. cubense tropical race 4 in the Cavendish banana
Source: PLoS One. 2024 Dec 2;19(12):e0313358. doi: 10.1371/journal.pone.0313358 (PMC11611109; doi:10.1371/journal.pone.0313358)
Supplement: S1 Fig — (PDF) [file pone.0313358.s001.pdf]

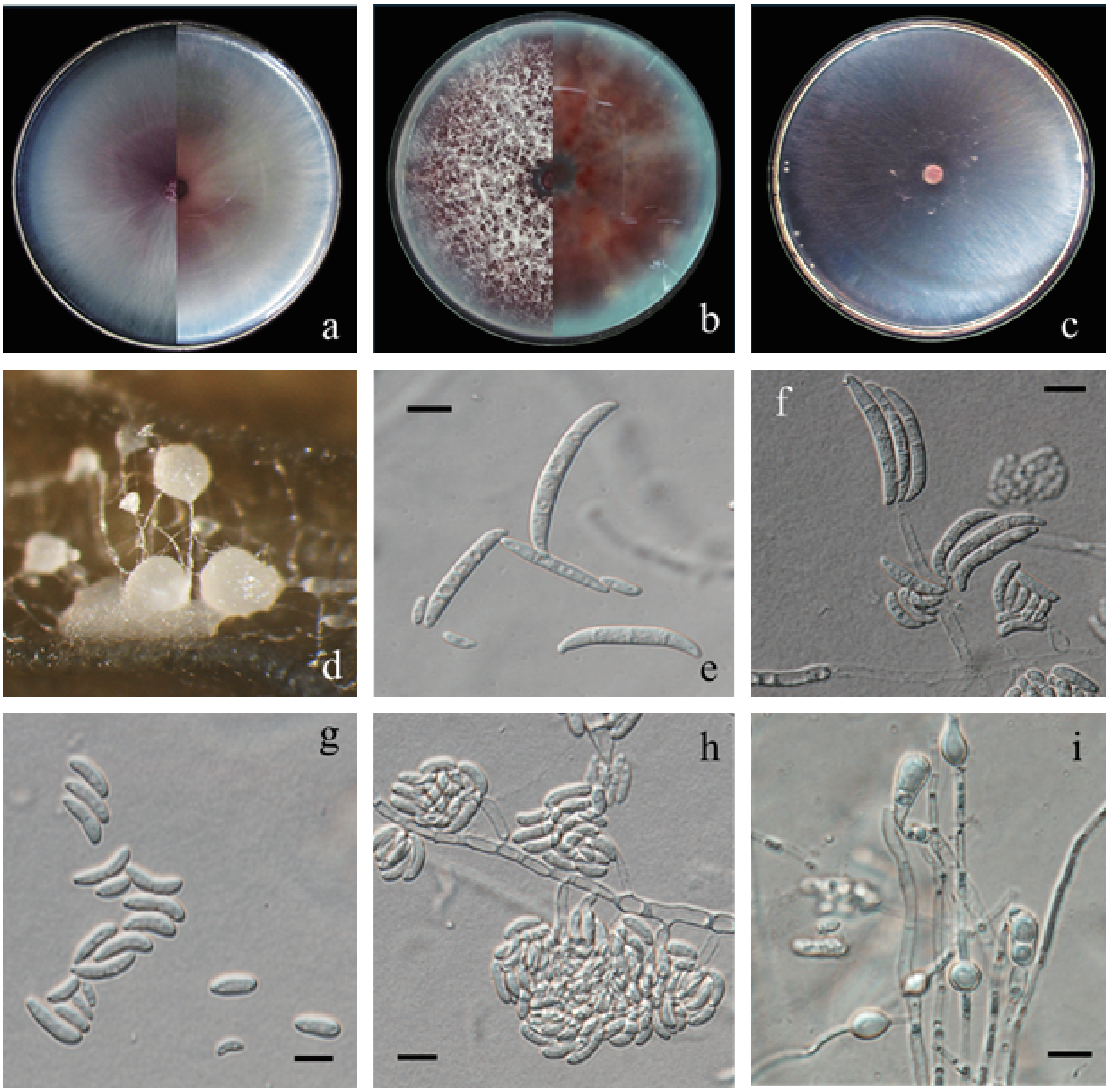

S1 Fig Morophology of 2718M (*Fusarium oxysporum* f. sp. *cubense*). Colony on a: Potato dextrose agar (PDA; obverse and reverse), b: Oatmeal agar (OA; obverse and reverse), c: Synthetic low-nutrient agar (SNA; obverse) after 7 days in the dark; d: sporodochia grown on carnation leaf agar (CNA); e-f: macroconidia; g-h: microconidia; i: chlamydospores. Scale bars = 10 μm.
