# Supplementary material for: Specific PCR primer designed from genome data for rapid detection of Fusarium oxysporum f. sp. cubense tropical race 4 in the Cavendish banana
Source: PLoS One. 2024 Dec 2;19(12):e0313358. doi: 10.1371/journal.pone.0313358 (PMC11611109; doi:10.1371/journal.pone.0313358)
Supplement: S2 Fig — (PDF) [file pone.0313358.s002.pdf]

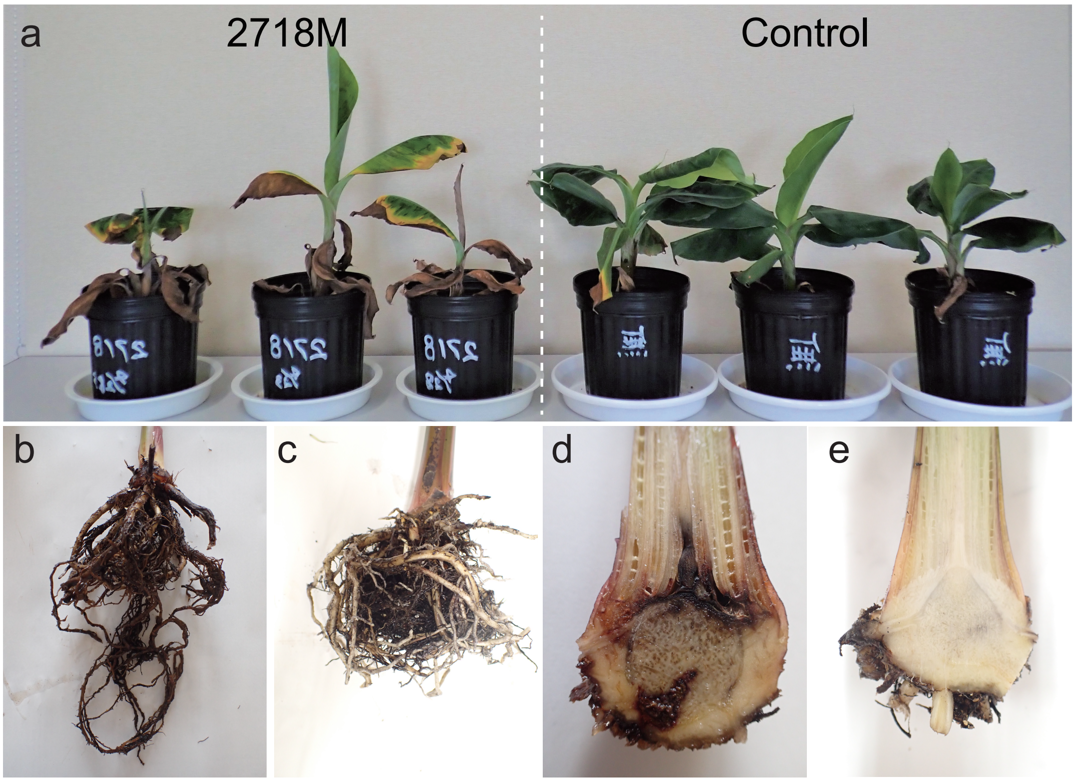


**S2 Fig The result of pathogenicity test of *F. oxysporum* f. sp. *cubence* tropical race 4 (2718M) to bananas (cv. dwarf Cavendish).** (a).Wilting symptoms 34 days after inoculation with 2718M (3 banana seedlings on the left) and control plants without symptom (3 banana seedlings on the right). (b). The roots of the inoculated plant. (c). The roots of the control plant. (d). A corm of the inoculated plant with blackish tissues and discolored tissues. (e). A corm of the control plant without symptom.

A pathogenicity test was conducted using a conidial suspension in sterilized water adjusted to $1 \times 10^7$ conidia/ml. Six Cavendish seedlings were used in this experiment, in which the roots of three seedlings were soaked in 500 ml of the conidial suspension for 3 h, then planted in pots with a 1:1 mixture of red ball earth and humus. A 5 g/L solution of NPK 8-8-8 was added as a chemical fertilizer. The remaining three seedlings were treated with sterilized water as a control. The treated and control plants were inoculated for 34 days at 25°C with an 8 h light/16 h dark cycle.
