# Supplementary material for: Specific PCR primer designed from genome data for rapid detection of Fusarium oxysporum f. sp. cubense tropical race 4 in the Cavendish banana
Source: PLoS One. 2024 Dec 2;19(12):e0313358. doi: 10.1371/journal.pone.0313358 (PMC11611109; doi:10.1371/journal.pone.0313358)
Supplement: S3 Fig — Lane M, marker; lane 1, Foc TR4; lane 2, Foc race 1; lane 3, Foc race 2; lane 4, MAFF103008; lane 5, MAFF235154; lane 6, MAFF240805; lane 7, MAFF237022; lane 8, MAFF243476; lane 9, MAFF727508; lane 10, MAFF306716; lane 11, MAFF744004; lane 12, MAFF305544; lane 13, MAFF240804; lane 14, MAFF243255; lane 15, MAAFF241054; lane 16, MAFF306313; lane 17, MAFF305937; lane 18, MAFF103036; lane 19, MAFF235105; lane 20, MAFF744088; lane 21, MAFF103054; lane 22, MAFF305606; lane 23, MAFF240327; lane 24, MAFF103059; lane 25, MAFF103051; lane 26, MAFF240102; lane 27, MAFF305543; lane 28, MAFF305608; lane C, ddH2O. (PDF) [file pone.0313358.s003.pdf]

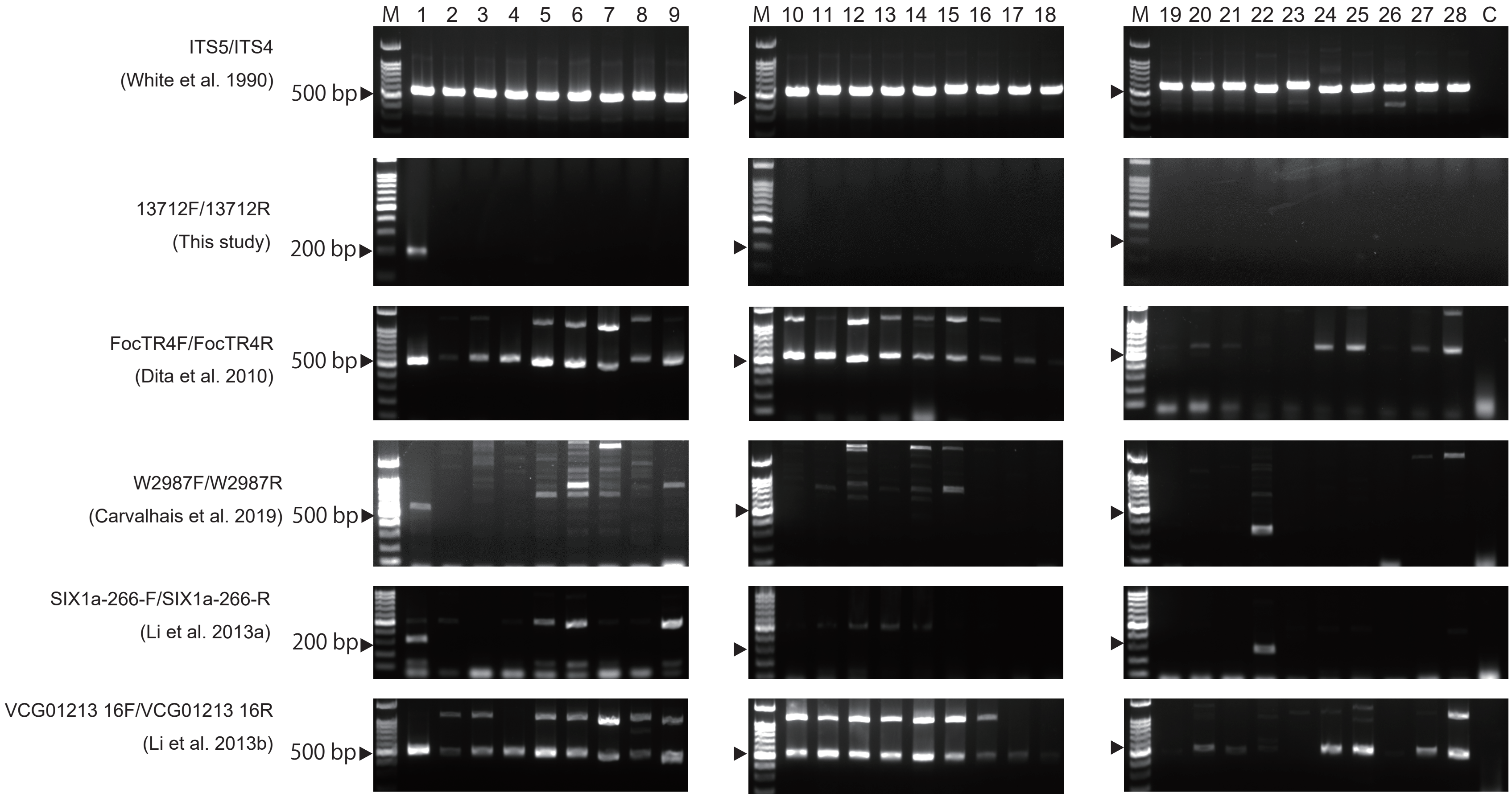

S3 Fig PCR detection of FocTR4 using specific primers sets: 13712F/13712R (this study), FocTR4F/FocTR4R (Dita et al. 2010), W2987F/W2987R (Carvalhais et al. 2019), SIX1a-266-F/SIX1a-266-R (Li et al. 2013a), VCG01213 16F/VCG01213 16R (Li et al. 2013b). Lane M, marker; lane 1, FocTR4;lane 2, Foc race 1;lane 3, Foc race 2;lane 4, MAFF103008; lane 5, MAFF235154; lane 6, MAFF240805;lane 7, MAFF237022; lane 8, MAFF243476; lane 9, MAFF727508; lane 10, MAFF306716; lane 11, MAFF744004;lane 12, MAFF305544; lane 13, MAFF240804; lane 14, MAFF243255; lane 15, MAAFF241054; lane 16, MAFF306313; lane 17, MAFF305937; lane 18, MAFF103036; lane 19, MAFF235105; lane 20, MAFF744088; lane 21, MAFF103054; lane 22, MAFF305606; lane 23, MAFF240327; lane 24, MAFF103059; lane 25, MAFF103051; lane 26, MAFF240102; lane 27, MAFF305543; lane 28, MAFF305608; lane C, ddH2O.
