# Supplementary material for: Specific PCR primer designed from genome data for rapid detection of Fusarium oxysporum f. sp. cubense tropical race 4 in the Cavendish banana
Source: PLoS One. 2024 Dec 2;19(12):e0313358. doi: 10.1371/journal.pone.0313358 (PMC11611109; doi:10.1371/journal.pone.0313358)
Supplement: S4 Fig — Isolate names are shown in each lane. The first lane of each panel (M) shows the marker sizes using the ExcelBand™ 100 bp DNA ladder (SMOBIO). The upper and lower panels show the amplification results for the ITS and 13712 regions, respectively. Isolates with red characters indicate amplification using primer set 13712F/13712R. Isolates with red dots indicate that isolates were identified as Foc TR4. (PDF) [file pone.0313358.s004.pdf]

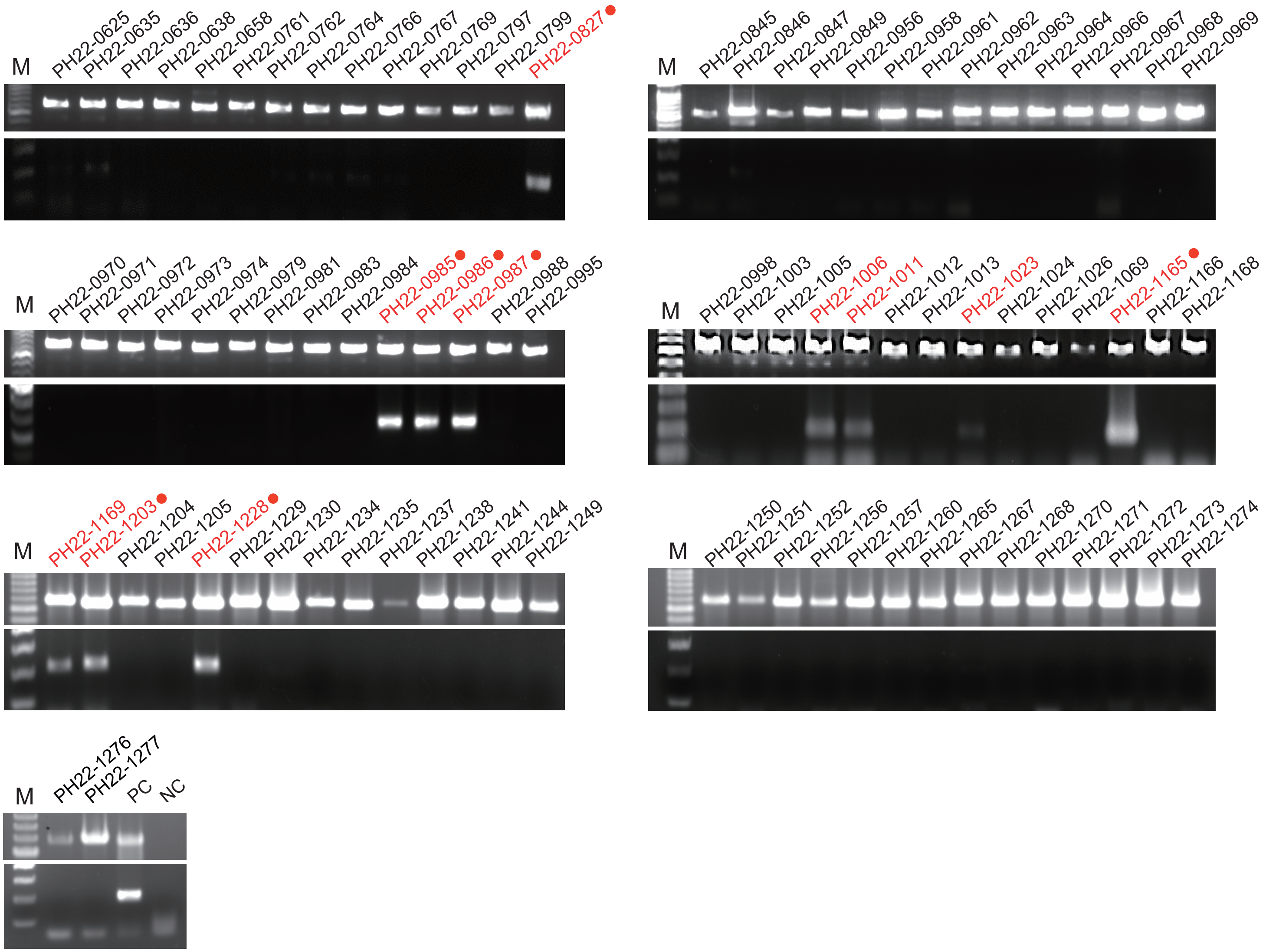

S4 Fig **PCR analysis of *Fusarium* spp. using the primer set 13712F/13712R. Isolate names were shown on each lane. Each panel's first lane (M) show marker sizes using the ExcelBand™ 100 bp DNA ladder (SMOBIO). The upper panel and lower panel are the result of the amplification of ITS and 13712 regions, respectively. Isolates with red character indicate that the target region was amplified by the primer set 13712F/13712R. Isolates with red dots indicate that isolates were identified as Foc TR4. Lane PC is positive control from strain 2718M (Foc TR4). Lane NC is negative control with ddH$_2$O.**
