# Supplementary material for: Specific PCR primer designed from genome data for rapid detection of Fusarium oxysporum f. sp. cubense tropical race 4 in the Cavendish banana
Source: PLoS One. 2024 Dec 2;19(12):e0313358. doi: 10.1371/journal.pone.0313358 (PMC11611109; doi:10.1371/journal.pone.0313358)
Supplement: Attachment — Submitted filename: Responce.docx [file pone.0313358.s010.docx]

**RESPONSE TO REVIEWERS**

Thank you for considering our manuscript (PONE-D-24-28376), now we revised sentences according to the reviewer’s advice. We particularly thank the reviewer for his/her incisive comment, which has helped us to make significant improvements to the scientific aspects of our study. Our responses to the comments are as follows.

**RESPONSE TO REVIEWE#1**

**Comments from Reviewer #1:**
*1. Expand the introduction, demonstrating the history of Fusarium wilt in bananas, for example, write a history since the discovery of banana wilt in Central America, going through the morphology, phylogenetic analysis (for example, how many lineages are there within Fusarium oxysporum f.sp. cubense). I found the authors' approach interesting, even designing the primers makes it clear that race 4 is a species called Fusarium odoratissimum.*
**Response**

Thank you so much for giving us advice for the introduction. I have followed your advice and made the following corrections.

**Line 34-38**

“However, banana production has been threatened by banana wilt disease caused by *Fusarium* *oxysporum* f. sp. *cubense* (Foc), a soil-borne pathogen. In the 20th century, the disease caused by Foc race 1 eradicated thousands of hectares of the Gros Michel banana in Central America. In response to this, the resistant variety, Cavendish banana was discovered and this variety became the dominant commercial banana worldwide.”

**Line 49-55**

“A recent study showed that although it is difficult to find differences in morphology from other races, Foc TR4 is independent of other lineages including Foc race 1 and Foc subtropical race 4 (STR4), based on phylogenetic analysis using four loci: calmodulin (*cmdA*), RNA polymerase second largest subunit (*rpb2*), translation elongation factor 1-alpha (*tef1*), and β-tubulin 2 (*tub2*) regions [6]. Therefore, Foc TR4 can be distinguished from other races by phylogenetic analysis.”

**Comments from Reviewer #1:**
*2. I recommend including in the material and methods photos of plants with symptoms of plants with wilt, or were the isolates obtained from a collection? 3. The authors do not mention the other isolates identified in the phylogeny, for example isolate PH 22-981 grouped with Fusarium fabacearum, the isolates grouped with Fusarium elaeidis, however, these results are not valued in the discussion.*

**Response**
We appreciate your suggestion that symptoms be shown. However, we do not have a picture of the original symptoms. So, instead of that, we added a result of the pathogenicity test against the Cavendish banana as supplemental data (S2 Fig) to show that 2718M strain is Foc TR4. The text below was added accordingly.

**Line 111-112**

“Foc TR4 isolates (2718M; S1 Fig) that confirmed its pathogenicity against the Cavendish variety and (S2 Fig),…”

***Comments from Reviewer #1:*** *4.The authors should value the phylogeny data, at no point do they mention the grouping of the isolates studied, please look at the phylogenetic tree and describe the data obtained.*
**Response**

Thank you so much for the valuable advice on the phylogeny of isolates. We agreed with your suggestion and added descriptions of the phylogenetic positions of our isolates in addition to that of Foc TR4 as below.

**Line 226-233**

“Other isolates were clustered with *F. elaeidis* (5 isolates; ML/NJ/MP = 73%/71%/84%), *F. fabacearum* (one isolate; ML/NJ/MP = -/-/78%), *F. trachichlamydosporum* (two isolates; ML/NJ/MP = 98%/98%/99%), and *F. triseptatum* (two isolates; ML/NJ/MP = 99%/99%/99%). Four isolates are independent of known species and closely related to F. glycines. One isolate, PH22-1069, of those, and three isolates, PH22-1006, PH22-1011, and PH22-1023, from other species complexes (not determined; Fig. 3) were detected using this primer set were not Foc TR4 (Figs 3 and 4, S4 Fig).”

***Comments from Reviewer #1:*** *5. What was the morphology of the isolates? Why not include a figure with the fungus cultures?*

**Response**
Thank you so much for your suggestion about the morphologies of the fungi. For 2718M, which was used for verification, we have included a photo of the isolate as supplemental data for reference (S1 Fig; **Line 111**). However, we did not include other isolates. This is because FocTR4 has been reported to be monophyletic in phylogenetic analysis, and it is sufficient to show the phylogenetic relationship to validate the primers.

***Comments from Reviewer #1:*** *6. In the discussion, the authors mention this information: "However, based on our comparison of primer specificities, the primer set 13712F and 13712R may offer better detection, although they are not as perfect as the other primers because some isolates (4 out of 79 non-Foc TR4 strains) that are from fields and not Foc TR4 were detected. Therefore, our primer successfully detected Foc TR4 in diseased plant tissue (Fig 5) and are expected to reduce the time and effort required for diagnosis". This worries me, specific primers should be perfect and the authors mention that they are not so perfect, they should justify this sentence better.*
**Response**

Thank you very much for the advice on the justification for the utilization of the developed primer set. We agree with your suggestion. The primers we developed would detect some other strains. However, they have proven to give better results when compared to primers developed previously. Therefore, we have revised the sentences as follows.

**Line 287-291**

“However, based on our comparison of primer specificities by PCR trials including other formae speciales, the primer set 13712F and 13712R offered better detection (Table 1; S1 Fig), although not complete because some isolates (4 out of 79 non-Foc TR4 strains) that are from fields and not Foc TR4 were detected. Furthermore, our primers successfully detected Foc TR4 in diseased plant tissue (Fig 5) and are expected to reduce the time and effort required for diagnosis.”

***Comments from Reviewer #1:*** *7. Although the research is important, I see that the manuscript needs to be improved.... I noticed that the main focus was only the design of the primers, but in addition, the authors obtained other results and this should be discussed.*
**Response**
We appreciate your suggestion. We agree with your advice. Without changing the main trend of validating the usefulness of the developed primers, we added to the results the phylogenetic relationships of the isolates obtained by phylogenetic analysis.

As described in the responses to suggestions 3 and 4, we added sentences as below.

**Line 226-233**

“Other isolates were clustered with F. elaeidis (5 isolates; ML/NJ/MP = 73%/71%/84%), F. fabacearum (one isolate; ML/NJ/MP = -/-/78%), F. trachichlamydosporum (two isolates; ML/NJ/MP = 98%/98%/99%), and F. triseptatum (two isolates; ML/NJ/MP = 99%/99%/99%). Four isolates are independent of known species and closely related to F. glycines. One isolate, PH22-1069, of those, and three isolates, PH22-1006, PH22-1011, and PH22-1023, from other species complexes (not determined; Fig. 3) were detected using this primer set were not Foc TR4 (Figs 3 and 4, S4 Fig).”

**RESPONSE TO REVIEWE#2**

***Comments from Reviewer #2:***
*1. Line 130: It’s better to provide a list of the 86 isolates with locations as well as the positive and negative reference strains that would be used during the PCR screening.*

**Response**

**Line 251**

We appreciate your suggestion. We agree with that we add the list of 86 isolates. We added a table (Table 3) which contains the result of detection from collected isolates, and their locations, and their phylogenetic positions.

***Comments from Reviewer #2:***
*2. Line 170: Please make sure Fig.1 legend for Fig. 1 or Fig. 2 (Line 187), and vice versa. Fig.2 just shows the locations of two primer fragments.*

**Response**

Thank you so much for your correction. We replaced Fig. 1 and Fig. 2 in the proper place.

**Line 177-180** Fig 1

**Line 198-199** Fig 2

***Comments from Reviewer #2:***
*3. Line 181: Even gene 13712 is specific to TR4, aliment of the sequence through NCBI in case of false positive result. No more illustration about the sequence of unique gene 13712 and how to design the sequence. Is there any another better one?*

**Response**

Thank you so much for your suggestion about the sequence of the target of the specific primers.

**Line 192**

We added the sequence data of the target region as supplemental data (S1 Data).

**Line 193-195**

We added the following sentence as the information on the primer set's design. “For a forward primer 13712F, GC content is 40%, and the annealing temperature is 53.2℃. For a reverse primer13712R, GC content is 26.9%, and the annealing temperature is 54.6℃.”

***Comments from Reviewer #2:***
*4. Line 242: The legend should mention each line referring to and whether the positive, negative, empty control went together with.*
**Response**
We appreciate your correction. We have reflected on this comment and we changed the explanation of Figure 5 about the positive and negative control as follows.

**Line 260-265**

Fig 5. Detection in plant tissues by PCR amplification. a Diseased (left) and healthy (right) corms of dwarf cavendish banana with and without inoculation of Foc TR4 (2718M). b Extracted DNA from healthy and diseased tissues. Lane M: 1 kb DNA ladder; Lane D: genome DNA from diseased tissue; Lane H: genome DNA from healthy tissue. c Detection of PCR amplicons using DNA templates from healthy and diseased tissues and mycelia of Foc TR4 (2718M). Lane M: 100 bp DNA ladder. Lane D: PCR amplicon from diseased tissue. Lane H: PCR amplicon from healthy tissue. Lane My: PCR amplicon from mycelia. Lane W: Negative control with ddH2O.

**RESPONSE TO REVIEWE#3**

***Response to Reviewer #3***
Thank you very much for recommending our paper for publication. We have revised the content of the paper in accordance with the advice of reviewers 1 and 2.
